# Head and neck cancer in Styria: An epidemiologic and clinical audit

**DOI:** 10.1007/s00508-019-01591-y

**Published:** 2020-01-15

**Authors:** Sarah M. Vasicek, Prisca Pondorfer, Clemens Holzmeister, Matthias Graupp, Thomas Weiland, Axel Wolf, Ulrich Moser, Dominik Wild, Dietmar Thurnher

**Affiliations:** 1 Department of Otorhinolaryngology, Head and Neck surgery, Medical University of Graz, Auenbruggerplatz 26, 8036 Graz, Austria; 2 Department of Otorhinolaryngology, Krankenhaus der Barmherzigen Schwestern Ried, Schlossberg 1, 4910 Ried im Innkreis, Austria

**Keywords:** Head and neck malignancy, Geographical distribution, Risk factor, TNM classification, Austria

## Abstract

**Background:**

The outcome of patients with cancer of the head and neck is significantly improved by increased interdisciplinary cooperation. The main focus of this study was a comparison of epidemiologic factors (age, sex, origin, staging) of patients with head and neck cancer in Styria, with those for patients throughout Austria.

**Methods:**

A retrospective data analysis of collected archived tumor board protocols of the Comprehensive Cancer Center (CCC) Graz included the patient’s age, sex, area of residence, TNM stage, reasons for inoperability, comorbidities and performance status by ECOG (Eastern Cooperative Oncology Group), was performed. This study focuses on 340 patients who presented with a head and neck malignancy for the first time.

**Results:**

In the period from January 2014 to December 2015 a total of 252 men (74.1%) and 88 women (25.9%) with malignant head and neck tumors, were presented in the tumor board for the first time. The mean age at diagnosis was 63.4 years. In 45.5% the patients already demonstrated advanced tumor stages (T4 = 27.9%, T3 = 17.6%). Most newly diagnosed neoplasms were cancers of the oropharynx (24.1%), larynx (19.4%) and oral cavity (18.8%) and 36.5% were considered to be inoperable. Curative and palliative treatments were initiated in 83.2% and 16.9%, respectively.

**Conclusion:**

The region of south Styria showed a higher incidence of T3 and T4 tumors of the oropharynx than the average Austrian population. Measures to increase awareness of this problem should be initiated to support general otorhinolaryngologists and general practitioners in detecting oropharyngeal cancers at an earlier stage.

## Introduction

Head and neck cancer is the ninth most common cancer worldwide, although different cancers vary in the frequency of occurrence globally [1]. The most common histopathological entity of malignant head and neck tumors is the squamous cell carcinoma with approximately 90% [2]. Tumors of this entity invasively expand and rapidly develop cervical lymph node metastases or even develop distant metastases, although local treatment has previously been effective [2]. Of the patients with cancer of the lip and oral cavity 10–30% subsequently develop second primary neoplasms of the upper aerodigestive tract [3, 4].

In western Europe cancer of the tonsils and oropharynx are the most common neoplasms of the head and neck [2, 5]. Data available from *Statistics Austria* confirmed 1225 new diagnosed malignant neoplasms of the lip, oral cavity and pharynx in the year 2014 in Austria [6]. More than 50% of diagnoses were made when the tumor had already reached a higher tumor stage [6]. Additionally, *Statistics Austria* published 291 new cases of diagnosed laryngeal carcinoma in the year 2014 [6]. In at least one third of all new cases the patients presented with advanced diseases (regionalized tumor stage: 33%, distant metastasis: 3%) [6]. Head and neck tumors account for 3% of the annual cancer neoplasms and cancer deaths in Austria [6].

There are various treatment regimens for malignant tumors in the head and neck region, including surgery, radiotherapy, chemotherapy and immunotherapy. These are used as single modalities or in combination. The anatomical extent of the disease is one of the major factors determining the appropriate treatment regimen and its prognosis. A higher TNM (tumor/nodes/metastasis) classification correlates with higher Union for International Cancer Control (UICC) stages and therefore with a worse prognosis. Treatment recommendations based on the histopathology and stage of the tumor, and the overall health of the affected patient, are given after discussion in a tumor board; however, with advanced tumor stages, treatment options become more limited, the outcomes become worse and treatment costs rise exponentially.

Since 2001 weekly tumor boards with a multidisciplinary panel of experts have taken place at the Department of Otorhinolaryngology, Head and Neck Surgery at the Medical University of Graz. The centre is part of the Comprehensive Cancer Center (CCC) Graz, covering 8 out of 13 districts in Styria and the City of Graz, which has a population of approximately 800,000 inhabitants.

Worldwide there are well-known differences in the regional distribution of lip, oral cavity, pharyngeal and laryngeal cancers [5]. In our department we had a clinical suspicion that specific regions of Styria had a higher incidence of T3 und T4 tumors. The purpose of this study was to determine if this suspicion could be confirmed with hard data. This would hopefully allow us to develop a strategy to improve the situation by developing targeted prevention programs and increasing awareness.

## Material and methods

This retrospective study was carried out at the Department of Otorhinolaryngology, Head and Neck Surgery, Medical University of Graz. The study was approved by the ethics committee of the Medical University of Graz and conducted according to the Declaration of Helsinki on Biomedical Research Involving Human Subjects (EK–Nb. 28-520 ex 15/16). The data were collected from the protocols of the interdisciplinary tumor board for head and neck cancer in the years 2014 and 2015. All patients with head and neck cancer (except cancer of the skin) with tumor stages pT1, pT2, pT3 and pT4 as well as T0 (CUP = carcinoma of unknown primary) were included. The tumors were staged according to the UICC TNM classification (7th edition UICC 2010).

The data collected included the following variables: date of birth, sex, age at first diagnosis, TNM stage at first diagnosis, place of residence within Styria, histological type and grading of the tumor according to the standard International Classification of Diseases for Oncology. Additionally, reasons for inoperability as well as comorbidities and the Eastern Cooperative Oncology Group (ECOG) performance status were recorded. The data were compared to the data for the whole of Austria, as provided by the official government database *Statistics Austria*.

In the years 2014 and 2015 a total of 460 patients suffering from head and neck cancer were discussed in the interdisciplinary tumor board. For this study, 120 of these patients were excluded: 96 patients due to previously diagnosed malignancies of the head and neck region, and 24 patients due to incomplete patient data. In part detailed patient information was not available because preliminary examinations including imaging and histologic results were performed in external hospitals and therefore no valid documentation could be found in the tumor board protocols.

## Limitations

A comparison of UICC staging was not possible due to missing N and M stage data in almost 50% of the cases provided by *Statistics Austria*. The number of patients with CUP in the head and neck region was not provided by *Statistics Austria*.

## Statistical analysis

Data analysis was performed using SPSS software, version 24.0; IBM SPSS Statistics, Chicago, IL, USA (c). Data are described as medians with ranges or means with standard deviations. Comparisons of continuous or categorical variables were performed with Student’s t‑test or the Mann-Whitney U‑test, and χ^2^-tests or Fisher’s exact test. The level for statistical significance was set at *P* = 0.05. For the comparisons between subgroups of patients regarding carcinoma localizations, TNM staging and patients’ geographical origin, Kruskal-Wallis analysis was used.

## Results

**Fig. 1:**
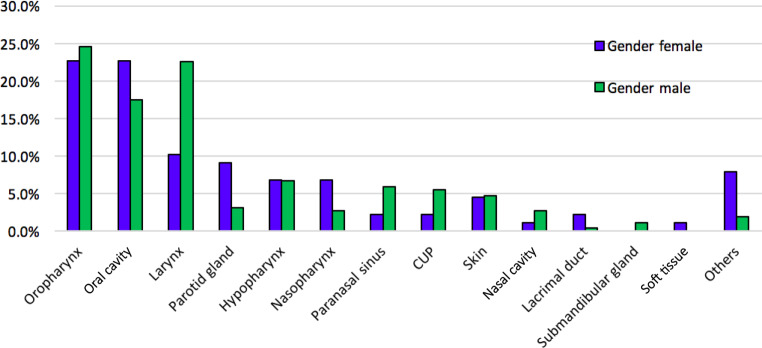
Distribution of localization of tumors in the head and neck region in male (*green*) versus female (*blue*) patients in Styria in 2014–2015.* CUP* carcinoma of unknown primary

**Fig. 2:**
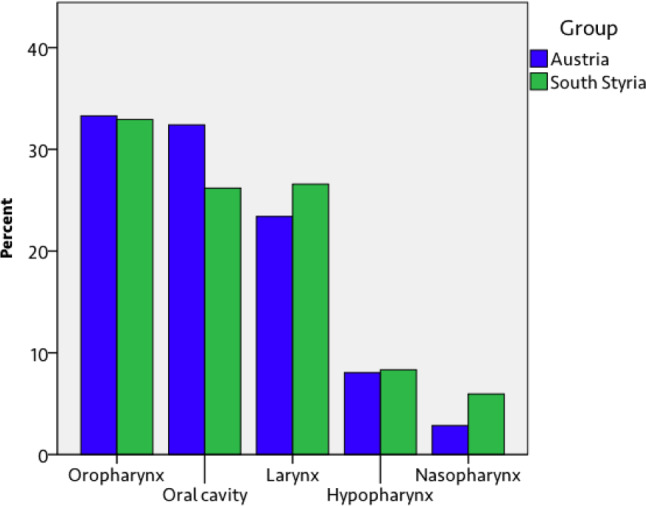
Distribution of the five most frequent tumor localizations in the head and neck region in Austria (south Styria *N* = 252, Austria *N* = 1790)

In the head and neck tumor board at CCC Graz, a total of 340 patients were registered as primary head and neck cancers (*n* = 144 in 2014, *n* = 196 in 2015). Of the 340 patients in this audit, 252 were male (74.1%) with an average age of 63.1 years (±11.4 years), and 88 females (25.9%) with an average age of 64 years (±13.2 years). The mean age at diagnosis was 63.4 years (±11.9 years) with a range of 22–98 years. No significant difference in age at first diagnosis was shown in patients in south Styria compared to the whole of Austria. A detailed distribution of the tumor localization presented is shown in Fig. 1. The most frequent neoplasms were observed in the oropharynx (24.1%) followed by lesions of the larynx (19.4%) and of the oral cavity (18.8%). Compared to the overall cohort of Austria, in Styria a slightly higher rate of laryngeal cancers and lower rate of lesions in the oral cavity were observed (Fig. 2). In the cohort p16 was determined in 74 out of 82 patients with oropharyngeal carcinoma and 25 (35%) patients were found to be positive.

Out of the 340 patients 95 (27.9%) were staged as T4 tumors and 60 (17.6%) cases as T3 tumors. Only 62 (18.2%) patients presented with T2, and 80 (23.5%) patients with T1. 22 (6.5%) patients were diagnosed as T0 (CUP) and 1 (0.3%) patient as TIS (Carcinoma in situ). Tumor staging was not available in 20 (5.9%) patients due to incomplete documented TNM-classification in the tumor board protocols. There was no significant difference in distribution within the T‑classification between women and men.

Men with T4 carcinomas presented at an earlier age than female patients (m: 67 years ± 9.8 years vs. f: 61 years ± 10.1 years; *p* = 0.019), but there was no significant difference between women and men in terms of age at first presentation in tumor stage T1, T2 or T3. This could also be shown in the total Austrian cohort. Female patients with T4 hypopharyngeal carcinoma (*p* = 0.016), T4 oropharyngeal carcinoma (*p* = 0.044) and T4 carcinoma of the oral cavity (*p* = 0.025) presented at a significantly higher age than male patients.

**Fig. 3:**
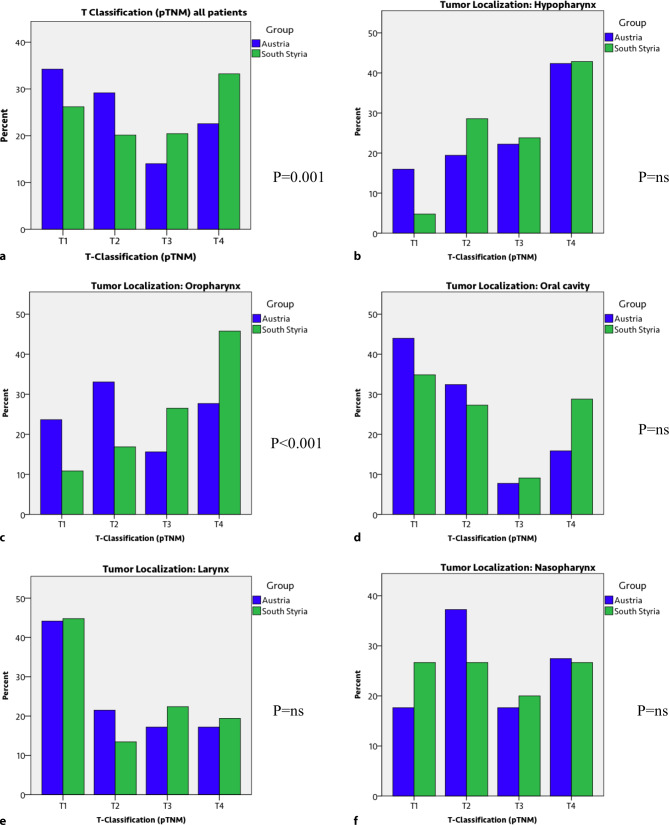
Distribution (T classification) of cancer diseases in the head and neck region in Austria and south Styria, in percent. *T1–T4* Tumor classification according to TNM UICC 7th edition, **a** Distribution of T-classification of all patients with head & neck carcinoma, **b** distribution of T-classification of patients with hypopharyngeal carcinoma, **c** distribution of T-classification of patients with oropharyngeal carcinoma, **d** distribution of T-classification of patients with oral cavity carcinoma, **e** distribution of T-classification of patients with laryngeal carcinoma, **f** distribution of T-classification of patients with nasopharyngeal carcinoma. *P* *p*-value, *pTNM* pathological tumor classification, *T1–T4* tumor classification according to TNM (tumor, node, meatsasis) classification UICC (Union International Contre le Cancer) 7th edition

**Fig. 4:**
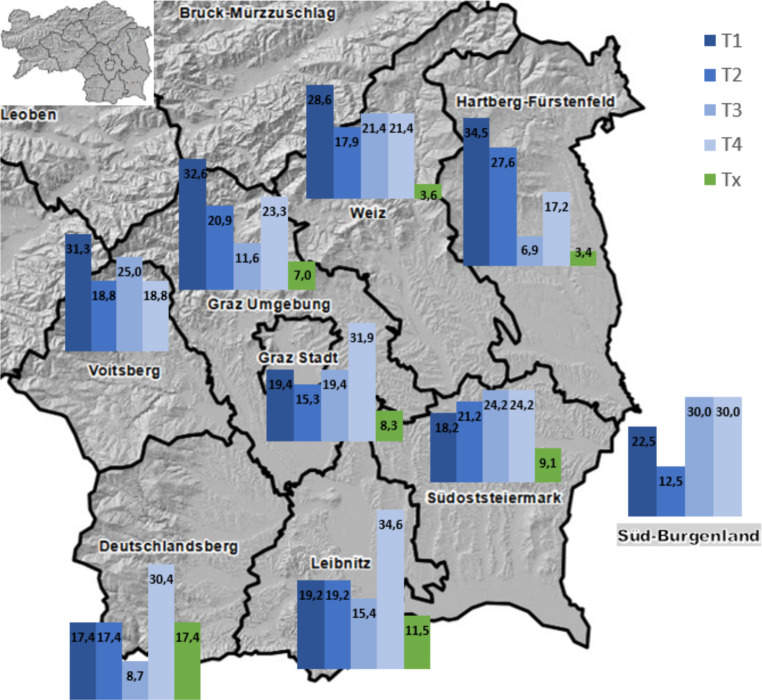
Catchment area of the Department of Otorhinolaryngology, Head and Neck surgery, Medical University of Graz, CCC Graz.The figure outlines the distribution of T-Classification in percentage (%) within each district in South Styria (*grey map*). *T1*–*T4* size or direct extent of the primary tumor, *Tx* tumor cannot be assessed, according to TNM (tumor, node, meatsasis) classification UICC (Union International Contre le Cancer) 7th edition

Fig. 3a–f show the distribution of the T classification within the tumor localizations for Austria and south Styria. There was a significant difference in T classification in the oropharynx (*p* < 0.001) between south Styria and Austria, and a slight difference was also observed between upper and lower south Styria (Fig. 4) but neither was statistically significant.

Tumors with higher stages at primary presentation were those of the paranasal sinuses (53.3% T4), the hypopharynx (39.1% T4) and the oropharynx (44.4% T4). In our study group, 47.4% of patients did not show cervical lymph node metastases (N0) at the time of the first presentation, while N3 staging was only observed in men (1.3%, *N* = 4). Of the patients 22 (6.5%, male *N* = 18, female *N* = 4) presented with a distant metastasis (M1) at the first staging. Most patients (88%) presented in good general health with an ECOG score of 0– 1. A high ECOG score, indicating a low physical performance status, significantly correlated with higher tumor classifications (66.7% with ECOG 4 were diagnosed T4, *p* < 0.001). Of the patients 160 (47.2%) did not undergo surgery. The most common causes for this were tumor localization and/or tumor extent (39%), comorbidities (27%) and/or poor general condition or advanced age (6.4%). In 15.6% no documentation regarding the operability could be found and 12.1% rejected the proposed surgical intervention.

In our cohort, 8 out of 12 patients with nasopharyngeal carcinomas received combined radiochemotherapy as first line treatment according to the NCCN (National Comprehensive Cancer Network) guidelines [7]. The result of the histopathological tissue examination of the biopsy was unclear at first. The final result showed the existence of a nasopharyngeal carcinoma. Another one underwent surgery (neck dissection, no tumor surgery) because of progressive lymph node metastasis and in another patient, elective lymphadenectomy was discussed but not performed. In one patient tumor debulking was performed under the aspect of a clivus chordoma, a nasopharyngeal carcinoma was later diagnosed histologically. In 8 (66.7%) out of 12 patients with nasopharyngeal cancer EBV (Epstein-Barr virus) tested positive. Despite the high-grade tumor diagnoses, treatment regimens with curative intent were initiated in 83.2% and palliative therapy in 16.9% of the cases. Information regarding cancer-specific therapies were not available for the total Austrian cohort.

## Discussion

This study was performed to evaluate the epidemiological and medical data of patients with malignant head and neck tumors in the federal province of Styria, to see if there were any correlations between general health status, tumor stage and area of residence. Our data showed a similar male to female ratio and age distribution that would be expected from other published data [6], adding to the validity of our study. An interesting point was that women with T4 carcinomas presented at a significantly higher age than men in our study. This is probably explained by men’s higher exposure to risk factors, such as smoking and alcohol, leading to the development of higher tumor burdens earlier in life. According to the official government data provided by *Statistics Austria*, our centre managed about 11% of the total of 3170 new patients in Austria with head and neck cancer in 2014–2015. Comparing these data sets overall, there was no significant difference in the distribution of the TNM classifications. When analyzing the data for South Styria we observed a higher percentage of high-grade tumor stages at first presentation compared to Austria as a whole, confirming our clinical suspicion of a higher tumor burden in this region. The particularly high rate (50%) of patients staged T4 on initial presentation from one district of South Styria could not be explained so far and requires further investigation. The reason for the higher rate of T4 carcinomas in two other districts (Leoben and Bruck) is explained by the fact that the hospital responsible for these areas only refers patients with advanced disease to our centre and manages all other patients locally. Unfortunately, the data of LKH Leoben’s local tumor board was not available for our current study. We have also been able to show a relatively high proportion of advanced tumor stages in the sites that are typically associated with late presentation. A third (27.9%) of our overall cohort had already been staged as T4, but within this, there was a higher proportion of T4 carcinomas of the paranasal sinuses (53.3%), hypopharynx (39.1%), and of oropharyngeal cancers (44.4%). Tumors in these localizations often cause only minimal or unspecific symptoms, leading to delays in visiting a specialist. We found that most patients were in very good general condition at the time of the first clinical visit, despite advanced disease; however, high ECOG performance scores correlated with high T‑classifications (66.7% with ECOG 4 were diagnosed T4).

**Fig. 5:**
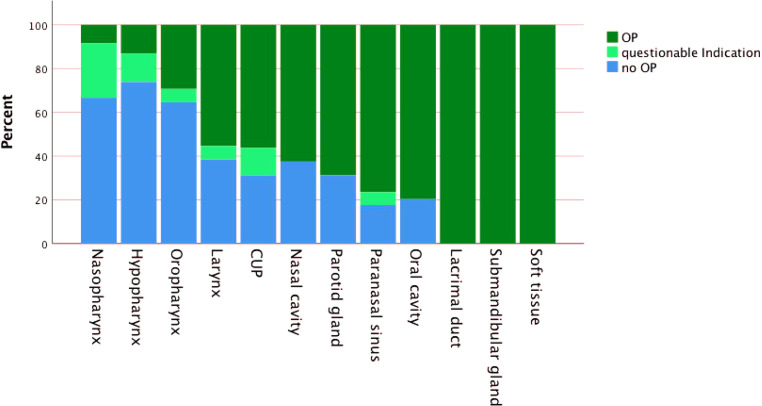
Surgical treatment of patients with head and neck cancer presented at CCC Graz, Medical University of Graz. *Blue* no surgery was recommended or carried out. *Light green* surgery options were discussed but finally no surgery was carried out. *Dark green* surgery was performed. *CUP* carcinoma of unknown primary, *OP* operation

In this study 88.6% of the patients presented with low ECOG (<2), although 37% were already considered inoperable. In most of these cases, the tumor size and its extensions were the most significant limiting factors for undergoing surgery. In many cases not only the size but also the localization of the tumors were limiting factors, which made an appropriate surgical remediation of the tumor disease impossible. The relation between tumor localization and operability is outlined in Fig. 5.

Overall, tumor localization and/or tumor extent (39%), comorbidities (27%), and poor general health or advanced age (6.4%) were the most common reasons not to undertake surgery in our cohort. Apart from this, 13.9% of patients rejected operative intervention, although it would have been possible from a medical point of view. Choi et al. mentioned that patients from lower socioeconomic levels usually present with higher grade tumor stages at time of primary presentation consequently have poorer long-term survival chances [8–10]. Austria is one of the most developed countries with excellent infrastructure in the health care sector. We do not feel that access to healthcare could explain the differences in advanced tumor stages, as healthcare insurance is provided to every individual. More important is probably the fact that lower socioeconomic levels are associated with lower education levels, hence leading to less health awareness. Choi et al. also mentioned that ignorance of early symptoms, fear of malignant diseases and emotional barriers are among the reasons for late first medical consultations [8]. Increased incidence of some head and neck cancers is also seen in residential areas with low health awareness and low numbers of physicians [11, 12]. As shown by Wyss et al. in developed countries, approximately 75% of lip, oral cavity and pharyngeal cancers can be related to alcohol consumption and tobacco smoking [13, 14]. The regions with the highest alcohol consumption rates are those of North America and certain regions of Europe, especially Central and Eastern Europe. Corresponding to this, oropharyngeal cancer incidence rates are highest in these regions [8]. Furthermore, Shield et al. showed that rates of human papillomavirus (HPV) related oropharyngeal cancer have been increasing in North America and Europe in particular [5] and HPV-related oropharyngeal cancers have a better prognosis as patients show longer overall survival [15]. A government funded vaccination program against the most common HR-HPV types, such as HR-HPV type 16, for children of both sexes has already been implemented in Austria since 2014 [16]. This promises a decline in HPV-induced anogenital cancers as well as oropharyngeal cancers [17].

Evidently early diagnosis influences the prognosis [18] and is essential for a positive outcome. It is therefore of importance to increase awareness for malignant diseases in the head and neck region. Health checks by general practitioners are available once a year free of charge for all adults in Austria; however, an ear, nose and throat (ENT) medical examination is not part of this screening program. If it would be included, at least for patients with risk factors, small symptomless tumors of the head and neck region could possibly be diagnosed at an earlier stage. Another measure to reduce head and neck tumor rates could be more education of the population regarding risk factors, such as smoking, alcohol consumption and HPV infections.

## Conclusion

Our clinical suspicion has been confirmed, namely that most patients within a specific area in south Styria present with advanced tumor stages, and that this results in poor treatment options and outcomes for these individuals. Further investigations are planned to see if specific types of lifestyle correlate with regional differences in the tumor burden in Styria. This would allow a more targeted approach in the application of preventative public health measures. Generally, our study confirmed that it would be desirable to increase public awareness through the media, and to improve the provision of comprehensive information for general practitioners and ENT specialists about malignant head and neck cancer and its risk factors.
